# Case Report: Metastatic duodenal hepatoid adenocarcinoma: a rare pathology with exceptional response to dual immune checkpoint blockade

**DOI:** 10.3389/fonc.2026.1896620

**Published:** 2026-09-30

**Authors:** Ranju Gupta, Darshan Lal, Rohit Walia, Shanth Goonewardene, Elizabeth Dellers, Kanchan Gupta

**Affiliations:** 1 Department of Hematology Oncology, Lehigh Valley Health Network, Allentown, PA, United States; 2 Department of Hematology Oncology, WellSpan Health, Reading, PA, United States; 3 Department of Radiology, Lehigh Valley Health Network, Allentown, PA, United States; 4 Department of Pathology, Lehigh Valley Health Network, Allentown, PA, United States; 5 Tufts University, Medford, MA, United States

**Keywords:** duodenum, durvalumab, hepatocellular carcinoma, hepatoid adenocarcinoma, immunotherapy, tremelimumab

## Abstract

Hepatoid adenocarcinoma (HAC) is a rare, malignant, extrahepatic tumor with hepatocellular differentiation. HAC is often associated with elevated serum alpha-fetoprotein (AFP) concentrations, which makes it difficult to distinguish from primary hepatocellular carcinoma (HCC). It is a difficult diagnosis based on histopathology, as the immunohistochemistry (IHC) profile often overlaps with hepatocellular carcinoma. HAC can arise from multiple organs, including the stomach, esophagus, colon, pancreas, duodenum, and peritoneum, with the stomach being the most common site. The treatment of HAC is not well defined, as randomized trial data is not available. The overall prognosis of metastatic HAC with standard combination chemotherapy is extremely poor. Here, we present a case of metastatic duodenal HAC that achieved a sustained complete response to chemotherapy-free dual immune checkpoint blockade, which, to our knowledge, represents the first published report of a sustained complete response to chemotherapy-free dual immune checkpoint blockade in metastatic duodenal HAC and suggests a potential approach that warrants further clinical investigation. We acknowledge that a degree of diagnostic uncertainty remains, as HAC and HCC can exhibit essentially identical morphologic and immunophenotypic features, making the distinction largely clinical.

## Introduction

Hepatoid adenocarcinoma (HAC) is a rare, malignant, extrahepatic tumor with hepatocellular differentiation (1). HAC was first described by Bourreille and colleagues in 1970 as an extrahepatic tumor producing alpha-fetoprotein (AFP) (2). Later, Ishikura et al. reported seven AFP-producing gastric adenocarcinomas and proposed the term hepatoid adenocarcinoma, as those extrahepatic tumors were producing AFP (3). HAC is an extremely rare neoplasm with an annual incidence of 0.58-0.83 cases per million people (4). HAC is often associated with elevated concentrations of serum alpha-fetoprotein. However, since only 84.4% of HAC cases report elevated serum AFP, it is considered a characteristic, but not a universal, marker of HAC. Though HAC can originate in multiple organs, including the stomach, esophagus, colon, pancreas, lungs, ovaries, duodenum, jejunum, peritoneum, gallbladder, and uterus, it most commonly occurs in the stomach (1). The symptoms of HAC are non-specific and depend on the site of origin. HAC pathogenesis and the mechanism of elevated serum AFP remain unclear. One theory is that the liver originates from the endoderm and develops from the primitive foregut during embryonic development. Thus, cancers originating from the gut may dedifferentiate into very early embryonic hepatocytes and produce AFP (1). HAC is generally more aggressive, has different clinicopathological characteristics, a poorer prognosis, and delayed diagnosis compared to typical HCC. Chen et al. revealed that a group with higher AFP expression had a higher frequency of liver metastasis and worse overall survival compared with those with lower AFP expression among patients with HAC (5). The most common sites of metastases are the lymph nodes and liver, followed by the lungs (6). Most patients are diagnosed at an advanced stage. Current literature suggests advanced stage at time of diagnosis and liver metastasis as the most common site of metastasis.. Somatic gene sequencing of HAC revealed TP53 (tumor suppressor gene) as the most common mutation (7). Immunohistochemistry (IHC) is widely used to help in the diagnosis of HAC, as HAC closely resembles HCC, making it difficult to differentiate between the two diagnoses. Su et al. (6) summarized 53 papers and revealed that HAC expresses several different markers, including Glypican-3 (100%), AFP (91.6%), HepPar-1 (38.1%), CEA (78.7%), CK7 (15.4%), CK18 (100%), CK19 (100%), CK20 (28.6%), α1-AT (91%), and CD10 (62.5%). HAC was more common in males than females, and most cases have been reported in Asian countries, especially China and Japan. The optimal management of HAC is not known because of the lack of data from randomized controlled trials. In early-stage HAC of the stomach, radical surgery is often curative (4). Cisplatin-based chemotherapy is considered to be an effective first-line systemic therapy for metastatic HAC, with clinical responses observed in 75% of patients (8). Thirteen HAC patients receiving palliative chemotherapy were described by Sun Kyung Baek et al. Fluoropyrimidine- and platinum-based chemotherapies were the most frequently used regimens (n = 7), followed by a combination of paclitaxel and cisplatin (9). Mei et al. reported a case in which pembrolizumab plus bevacizumab was used as a second-line treatment for metastatic HAC (after the patient progressed on chemotherapy) and reported a longer progression-free survival (PFS) of 14 months compared to conventional chemotherapy (10).

## Case presentation

A 63-year-old African American male patient presented to the emergency department (ED) with symptomatic severe microcytic anemia, with a hemoglobin of 5.8 g/dL(gram/deciliter) and ferritin of 5 ng/mL(nanogram/milliliter). He denied having blood in his stools or black-colored stools. He received blood transfusions and underwent upper endoscopy (EGD) and colonoscopy, both of which were negative for acute pathology. His past medical history was significant for well-controlled diabetes. He was a nonsmoker and had no significant history of alcohol use. He had no known history of chronic liver disease, cirrhosis, hepatitis B infection, or hepatitis C infection. CT abdomen/pelvis showed SMV and portal vein thrombosis (**Figure 1**) but no radiological features of cirrhosis, portal hypertension, or a dominant primary hepatic mass. Further workup with liver MRI revealed innumerable liver lesions consistent with metastatic disease (**Figure 2**). His serum AFP was >50,000 ng/mL. Liver mass biopsy was positive for Glypican-3, HepPar-1, and AE1/AE3 and was reported to be consistent with hepatocellular carcinoma (**Figure 3**). While waiting to start systemic treatment, the patient presented to the ED (about 6 weeks from the initial admission) with nausea and vomiting and was found to have severe anemia again, with a hemoglobin of 5 g/dL. He underwent repeat EGD, which showed a friable 3 cm bleeding mass in the duodenal bulb that was causing partial obstruction. Repeat CT abdomen showed gastric distension and new lymphadenopathy around the duodenum. Due to persistent symptoms of partial gastric outlet obstruction, he underwent distal gastrectomy with Roux-en-Y bypass surgery. Final pathology from the duodenal mass was consistent with the liver mass biopsy based on IHC positivity for Glypican-3, HepPar-1, and AE1/AE3 and negativity for CK7, CK20, TTF-1, and Arginase (**Figure 3**). Due to the complexity of the case, the pathology specimen was sent to two outside institutions for review. Both the reviewers rendered a descriptive diagnosis of hepatoid carcinoma based on the morphology and immunostaining profile described above but noted that since the morphology and immunophenotype of hepatoid adenocarcinoma and hepatocellular carcinoma are essentially identical, the primary site would need to be determined clinically. Recent studies have suggested SALL4 (Spalt-like transcription factor 4) (11) for this differential diagnosis, with inconclusive results. SALL4 immunostaining was not performed because the diagnostic evaluation had been completed before its potential utility in this differential diagnosis was recognized. Clinically, hepatocellular carcinoma rarely presents with a discrete primary lesion in the duodenal bulb and subsequently metastasizes to the luminal gastrointestinal tract. In this case, the presence of a bleeding obstructing duodenal mass, concordant histopathology between the duodenal and hepatic lesions, regional lymphadenopathy surrounding the duodenum, and the absence of underlying cirrhosis or chronic viral hepatitis favored a duodenal primary tumor with hepatic metastases. Nevertheless, given the substantial morphologic and immunophenotypic overlap between HAC and HCC, a degree of diagnostic uncertainty remains, and complete exclusion of metastatic HCC is not possible. Meanwhile, his serum AFP levels continued to rise, peaking at 35 million ng/mL. Next generation sequencing from the final specimen revealed a TP53 mutation, microsatellite stable (MSS) disease, and TMB of 4 mut/Mb (mutations per megabase). After discussing the diagnosis, prognosis, and available treatment options with the patient, a decision was made to proceed with combination immunotherapy consisting of tremelimumab and durvalumab. As described by the authors of the HIMALAYA study (12), the patient received one dose of tremelimumab and durvalumab combination therapy followed by durvalumab (12). After 2 months of treatment, his AFP decreased to 142 ng/mL, and it normalized completely (<1.8 ng/mL) after 6 months of treatment. The patient continued to show a response to this treatment, with subsequent scans showing improvement in the liver lesions (**Figure 4**). After the patient completed two years of treatment and scans did not show any active disease; his treatment was discontinued (**Figure 4**). He had no autoimmune side effects from immunotherapy. He remains without evidence of recurrence one year after discontinuation of treatment. The overall chronology of diagnosis, treatment, AFP kinetics, radiographic response, and long-term follow-up is summarized in **Figure 5**.

**Figure 1:**
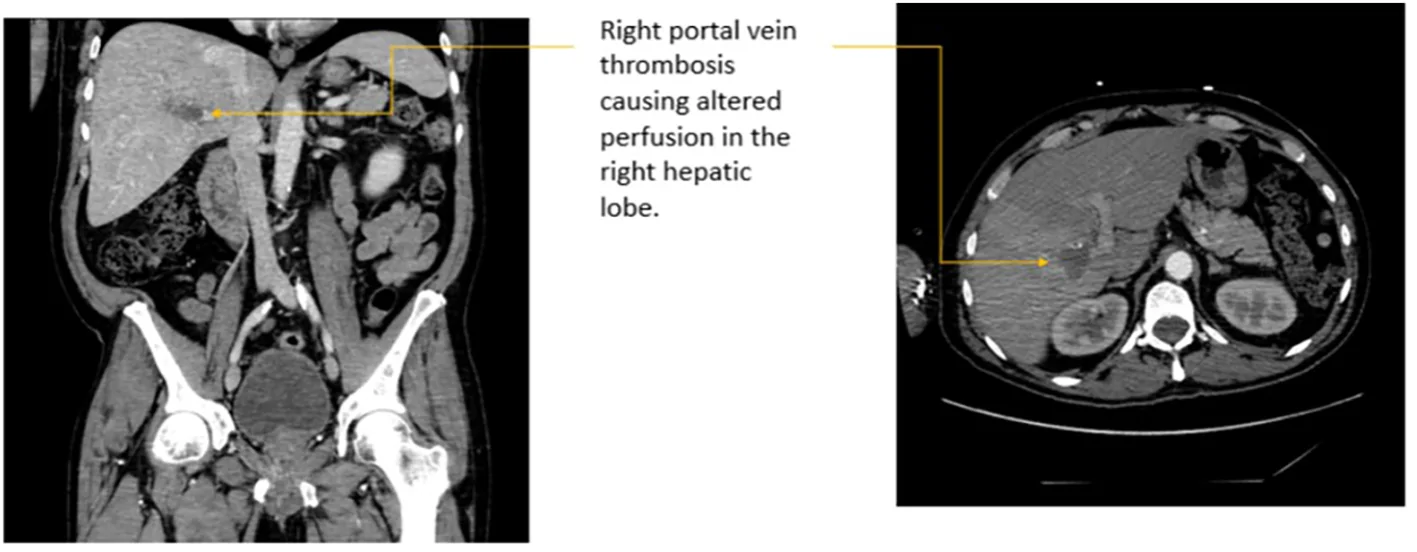
Initial pretreatment CT scan of the abdomen and pelvis. Multiple liver metastases are suspected but are not well delineated on computed tomography imaging.

**Figure 2:**
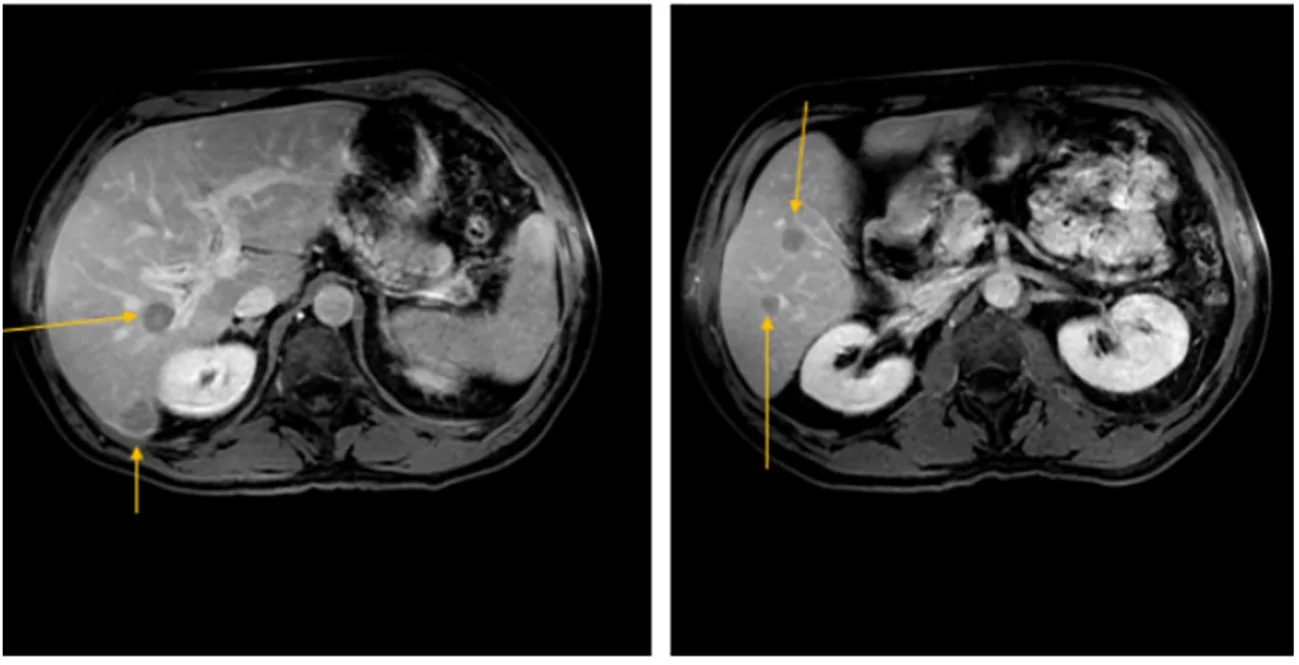
Initial pretreatment MRI of the liver. Magnetic resonance imaging confirms the presence of multiple liver metastases.

**Figure 3:**
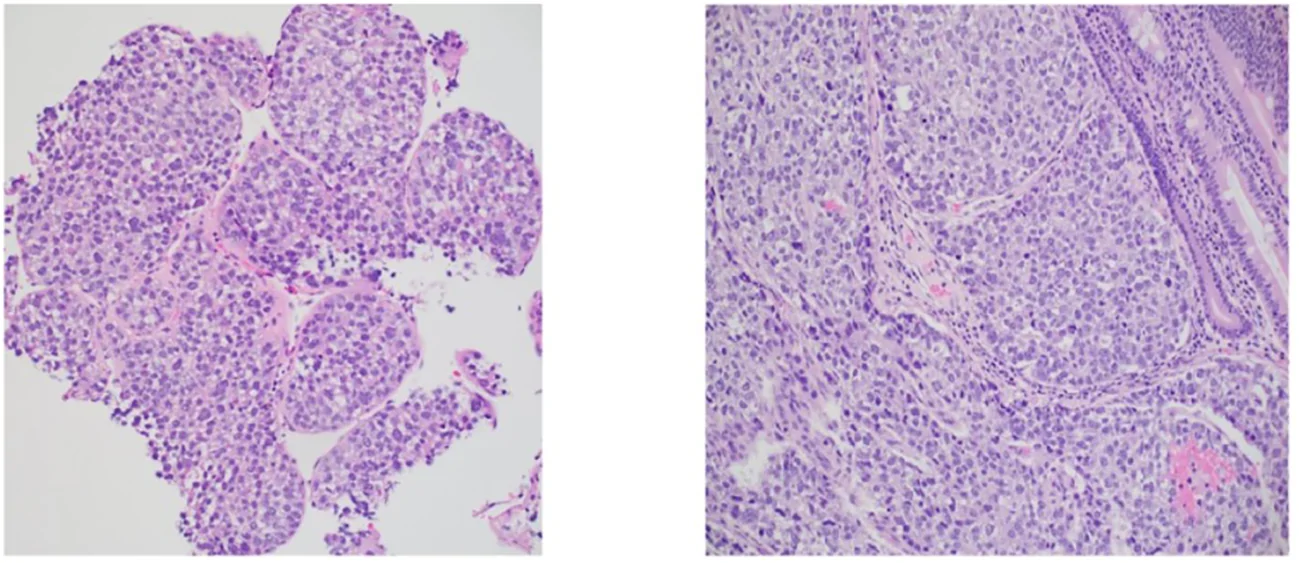
Histopathologic comparison of liver and duodenal lesions. Liver biopsy specimen (left) and duodenal tumor specimen (right) demonstrate morphologic similarity between the lesions. Hematoxylin and eosin (H&E) stain, original magnification ×200.

**Figure 4:**
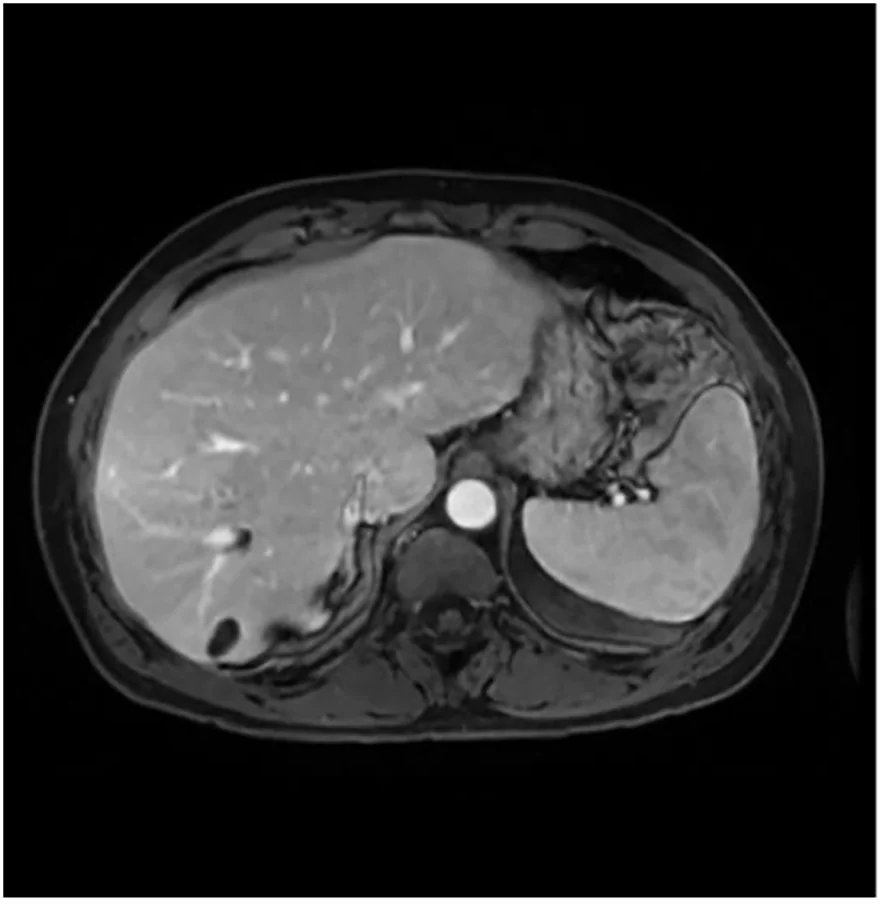
Post-treatment MRI of the abdomen. Follow-up magnetic resonance imaging demonstrates no enhancement of the treated liver metastases, consistent with complete radiographic response and no evidence of viable disease.

**Figure 5:**
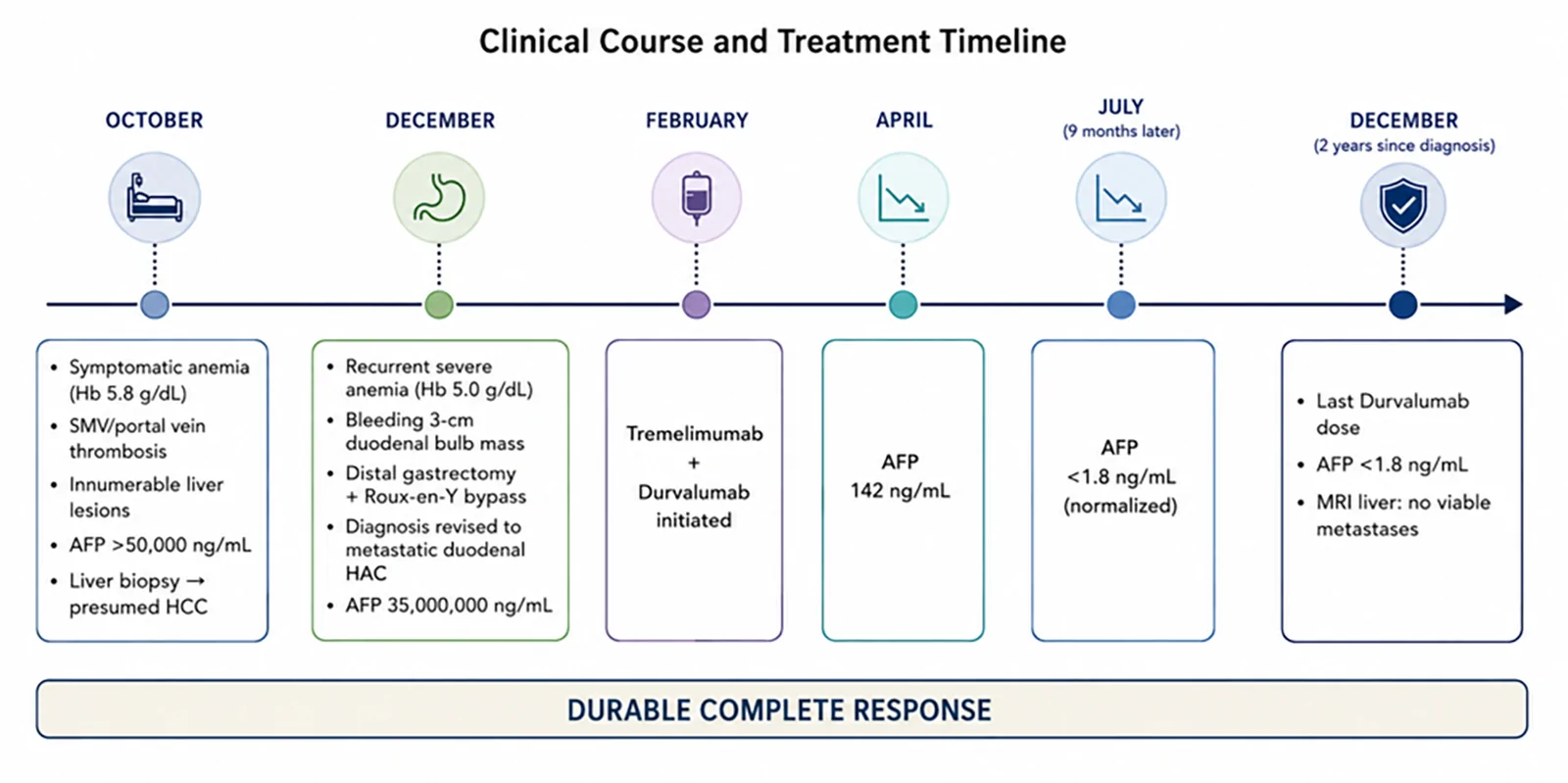
Clinical course and treatment timeline. Timeline depicting the patient's initial presentation, diagnostic evaluation, treatment with tremelimumab plus durvalumab, serial alpha-fetoprotein (AFP) response, and long-term outcome. AFP decreased from >50,000 ng/mL at presentation to normalization (<1.8 ng/mL) following therapy, with a durable complete response and no viable metastatic disease identified on follow-up imaging at 2 years.

## Discussion

HAC is a rare, aggressive, and extrahepatic adenocarcinoma that mimics HCC. The challenge lies in making the correct diagnosis, as it closely resembles HCC pathologically and, in the majority of cases, produces AFP. Once the diagnosis is made, there are no established guidelines to treat this aggressive cancer. Multiple case reports and case series have suggested an overall poorer prognosis for patients with HAC compared to HCC. Since gastric HAC is the most common type of HAC, most treatment data are extrapolated from gastric HAC. 5-fluorouracil- and cisplatin-based chemotherapies are the standard treatments in adjuvant or metastatic gastric HAC, with cisplatin being the preferred agent (13). Fujimoto et al. reported HER2 positivity in 25% of cases of gastric HAC (n = 16), and the addition of trastuzumab to chemotherapy in these cases has been recommended (14). Arakawa et al. reported a case of excellent response to monotherapy with ramucirumab (15). Similarly, progression-free survival of 14 months was reported in a case treated with pembrolizumab and bevacizumab as second-line treatment for gastric HAC (10). Li et al. reported a case of locally advanced, non-metastatic gastric HAS treated with neoadjuvant S1 along with oxaliplatin and sintilimab for 3 cycles, making the disease resectable (16, 17). Philip Haddad presented, in abstract form, a pooled analysis of 141 patients with gastric HAC at ASCO 2025. With male preponderance, the median age was 63 years, and 56% of cases were metastatic, with the liver (91%), lungs (23%), and peritoneum (14%) being the most common sites of metastases. Unlike previous data, the median OS was 20 months. He confirmed that platinum-based regimens were superior to non-platinum-containing regimens, with a median OS of 16 vs. 4 months, respectively. Age, sex, gastric location, size, and AFP levels did not impact OS. In comparison, duodenal HAC, which is rarer and has limited available data, has a dismal prognosis. In a case series of 42 patients with intestinal HAC (IHA), only 5 patients had duodenal HAC, and the majority of cases were colonic. The 1-year PFS and disease-specific survival (DSS) of IHA stratified by stages I-II, III, and IV were 66.7%, 50%, and 0%, respectively. All 21 patients with metastatic IHA at the initial diagnosis had died by the first year (18). The response to chemotherapy remains poor as well, and therefore, there is interest in targeted therapies. Next-generation sequencing in HAC reveals multiple alterations. Whole-genome sequencing of 38 gastric HAC patients showed TP53 mutation in 66% of cases. In the same cohort, Zhao et al. found that 55.26% of HAC patients had at least one clinically actionable alteration, including ERBB2, FGFR1, CDK4, EGFR, MET, and MDM2 amplifications and BRCA1/2 mutations (19). Additionally, 10.53% of patients had TMB >10 mut/Mb (20). Another study also reported TP53 as the most common mutation in HAC, along with microsatellite instability (MSI) and HER2 amplification (20).

It is unclear why our patient with duodenal HAC responded to the combination of durvalumab and tremelimumab despite having no actionable mutation on NGS other than a TP53 mutation. While we do not have a definitive answer, some insights can be drawn from the mechanism of action of combination immune checkpoint inhibitors (ICI) in HCC.

The most frequently identified mutations on NGS in HCC include TERT promoter (50–60%), TP53 (20–40%), CTNNB1 (15–40%), ARID1A (10–20%), AXIN1 (5–10%), and ARID2 (5–10%) (21). Conventional biomarkers used to predict response to ICIs, such as high PD-L1 expression, high tumor mutational burden (TMB), and microsatellite instability (MSI), are often absent in HCC. Despite this, HCC has demonstrated meaningful responses to combination immunotherapy with VEGF inhibitors or CTLA-4 inhibitors. As described by the authors of the HIMALAYA study (12), CTLA-4 inhibits the priming and activation of lymphocytes early in the antitumor immune response, whereas PD-L1 modulates immune responses within the tumor microenvironment downstream of lymphocyte activation. Therefore, blockade of CTLA-4 with tremelimumab promotes the proliferation and expansion of CD4+ and CD8+ T cells. These expanded T-cell clones are subsequently maintained by ongoing PD-L1 blockade with durvalumab (22). This complementary mechanism may partly explain the exceptional response observed in our patient despite the absence of traditional predictive biomarkers.

Our patient presented with *de novo* metastatic duodenal HAC with liver metastases. NGS revealed only a TP53 mutation. He demonstrated a sustained complete response to combination immunotherapy with durvalumab and tremelimumab and remains without evidence of disease more than three years after diagnosis. Given that metastatic duodenal HAC is associated with dismal prognosis with most reported cases demonstrating an overall survival of less than one year, this outcome is particularly noteworthy. Although the biological basis for this response remains unclear, HAC may share molecular and immunologic characteristics with hepatocellular carcinoma that could confer sensitivity to immune checkpoint inhibition. To the best of our knowledge, this appears to be the first published report of a sustained complete response to chemotherapy-free dual immune checkpoint blockade in metastatic duodenal HAC. While conclusions regarding efficacy cannot be drawn from a single case, this observation suggests potential of this treatment strategy. Further clinical investigation is warranted to better define the role of immunotherapy in this rare malignancy and to identify biomarkers predictive of treatment response.

A limitation of this report is the inability to definitively distinguish hepatoid adenocarcinoma from metastatic hepatocellular carcinoma on morphological and immunophenotype grounds alone. Although the overall clinical presentation favored a duodenal primary tumor with hepatic metastases, the substantial overlap between these entities prevents absolute diagnostic certainty. Nevertheless, regardless of the favored diagnosis, the durable complete response observed with dual immune checkpoint blockade remains clinically noteworthy.

## Data Availability

The original contributions presented in the study are included in the article/supplementary material. Further inquiries can be directed to the corresponding author.
